# Ten quick tips for spatial transcriptomics analysis

**DOI:** 10.1371/journal.pcbi.1014757

**Published:** 2026-09-15

**Authors:** Nagomi Kurogi, Koki Shimbara, Tatsuya Koreeda, Koki Tsuyuzaki

**Affiliations:** 1 Graduate School of Medicine, The University of Tokyo, Tokyo, Japan; 2 BaraPhaSilico, Ibaraki, Japan; 3 CLINIC FOR Group, Tokyo, Japan; 4 Data Science Core, Chiba University, Chiba, Japan; Montreal, CANADA

## Abstract

Spatial transcriptomics (ST) enables genome-wide gene expression profiling while retaining spatial context within tissue sections. Since the foundational work by Ståhl *et al*. in 2016, the field has expanded rapidly, with diverse platforms now spanning sequencing-based (e.g., Visium, Visium HD, Slide-seq, Stereo-seq, and Seq-Scope) and imaging-based (e.g., MERFISH, Xenium, and CosMx SMI) approaches. The breadth of platforms, data structures, and computational tools, however, can be daunting for newcomers. Here, we present ten quick tips spanning the entire ST research workflow: whether ST suits a given biological question, how to select a platform aligned with study objectives, how to understand and process ST data, and which software tools to employ for analysis and visualization. We further discuss interpreting spatial patterns in biological context, integrating complementary modalities such as single-cell RNA sequencing and spatial proteomics, and leveraging public datasets and sharing results. Finally, we highlight current limitations of ST, particularly the challenge of reconstructing three-dimensional tissue architecture from serial tissue sections. This review provides biologists, bioinformaticians, and clinician-scientists with a concise, platform-neutral roadmap for incorporating ST into research, from experimental design to biological discovery.

## Author summary

Spatial transcriptomics (ST) lets researchers measure gene expression while preserving the tissue location of each measurement. Where a cell sits, and which cells surround it, shape how it behaves—information that is lost when tissue is dissociated. Despite the growing popularity of ST, there is currently no concise, practical guide for researchers new to the field, for whom the diversity of platforms, data formats, and computational tools can be overwhelming. Here, we distill essential knowledge into ten quick tips covering the entire ST research workflow—from deciding whether ST is the right approach for a given biological question, through selecting an appropriate experimental platform, to analyzing and interpreting the resulting data. We also discuss integrating ST with other omics data and highlight current limitations that users should be aware of. Our goal is to help researchers across disciplines—including oncology, neuroscience, and developmental biology—design rigorous ST experiments, avoid common pitfalls, and generate reproducible, biologically meaningful insights.

## Introduction

Spatial transcriptomics (ST) comprises a family of technologies that measure gene expression in tissue sections while preserving spatial coordinates. In contrast to dissociation-based approaches such as single-cell RNA sequencing (scRNA-seq), ST links molecular measurements to tissue architecture, enabling researchers to ask where cell types, gene-expression programs, and disease-associated niches occur within their histological context [1].

ST is rapidly establishing itself as a tool for relating tissue structure to function across fields including oncology [2], neuroscience [3], and developmental biology [4]. As in other omics assays, the questions an ST study can answer are constrained well before analysis begins—but these constraints bind unusually tightly here: tissue preservation and handling, the spatial resolution and transcriptome coverage of the chosen platform, the available histological context, and the number of biological replicates jointly determine which spatial hypotheses are answerable. What is specific to ST is the statistical structure of the resulting data: neighboring measurements are spatially autocorrelated rather than independent, and the many spots in a section do not substitute for biological replication. Planning therefore means reasoning explicitly about these spatial and statistical properties from the outset, not only about cost or throughput.

In this article, we present ten quick tips as practical guidance for researchers employing ST, covering the entire research workflow from platform selection and data understanding to biological interpretation, multi-omics integration, and recognition of technical limitations.

## Tip 1: Confirm that spatial information is essential before committing to ST

Although ST is an innovative alternative to conventional experimental approaches such as bulk RNA-seq, it is not necessarily the optimal method for every research question. Before initiating a study, ask: is spatial information truly indispensable for addressing the biological question at hand? ST is most valuable when the spatial arrangement of gene expression is itself part of the question—for example, consider whether the overall question requires information on the organization of cell types, tissue architecture, local microenvironments and niches, signaling gradients, or cell–cell interactions interpreted in their tissue setting [2,4]. ST experiments are relatively costly per assay, and the resulting data are complex, requiring specialized expertise and time to analyze [5]. If the objective can be achieved with established methods such as scRNA-seq, immunohistochemistry (IHC), or in situ hybridization (ISH), consider whether ST is cost-effective [6].

This is especially relevant for large-scale screening, where ST throughput and cost remain limiting. ST is best suited to investigations seeking deep insight from a limited number of precious specimens—for example, dissecting microenvironment architecture in specific disease models [7]—whereas evaluating a predefined biomarker across hundreds of clinical specimens is typically far more efficient with IHC. Cost and throughput are real constraints, but they should be weighed as one consideration among several rather than treated as the primary criterion.

Finally, define the comparison and replication strategy before generating data. Decide which groups will be compared (e.g., disease versus control, or across anatomical regions) and build in biological replication at the inferential level—patients, animals, or samples—not the many spots within a single section (Tip 4). Decide also how much tissue to profile: small cores or tissue microarrays raise throughput but capture limited spatial context, whereas large sections preserve architecture at the cost of fewer samples and higher per-sample cost. In case–control designs, balance conditions across slides and batches, or randomize their placement, so that biological contrasts are not confounded with technical ones (Tip 5). Pilot experiments and sample-size reasoning here help establish whether an ST study can realistically support its conclusions.

## Tip 2: Choose an ST platform that fits your biological question and resources

**Fig 1:**
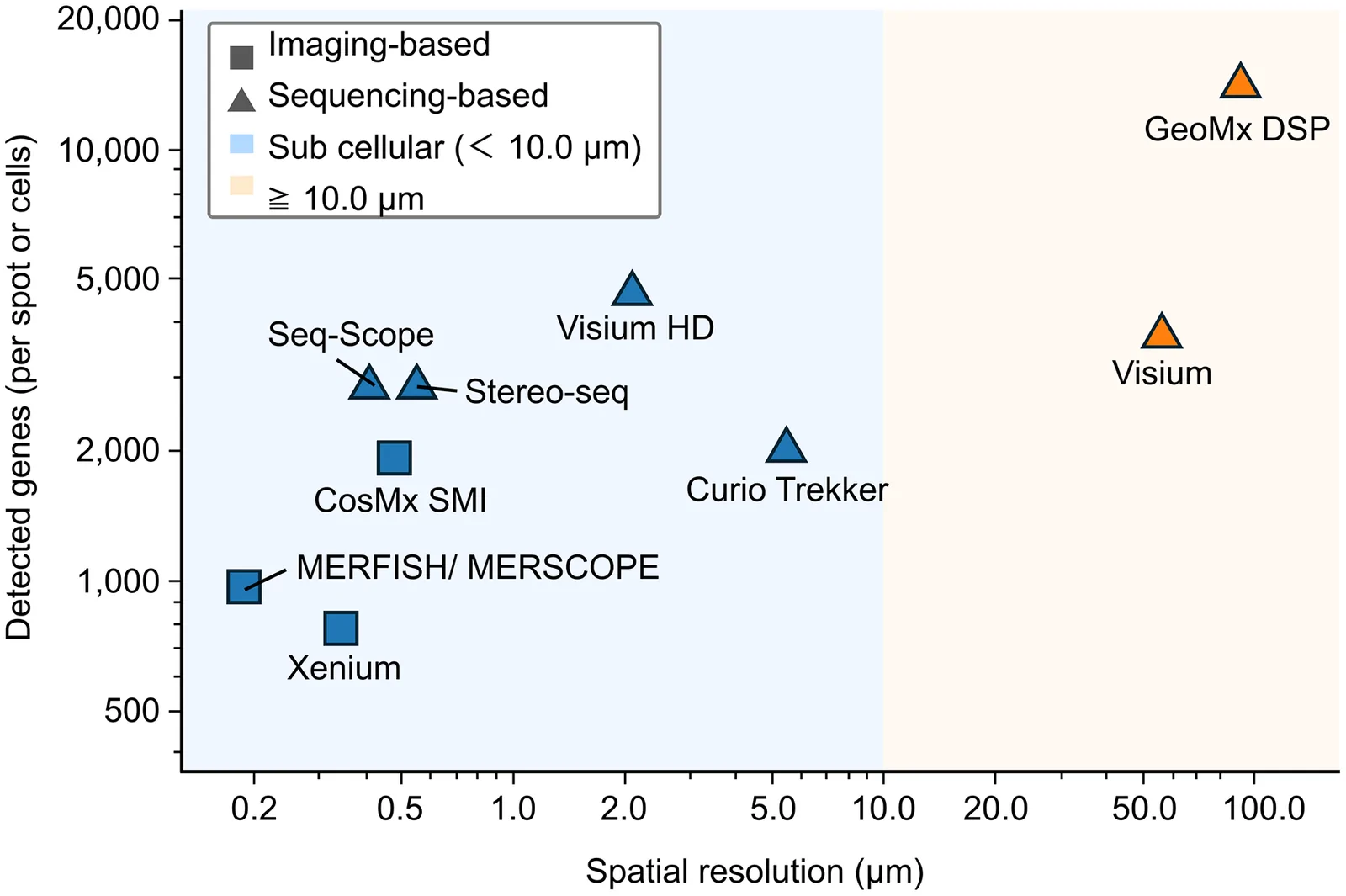
Spatial transcriptomics platforms compared by spatial resolution and transcriptome coverage. Imaging-based platforms and sequencing-based platforms are plotted by spatial resolution (x-axis, µm) versus the number of genes detected per spot or cell (y-axis). The same two-family classification is used in Table 1. Representative imaging-based methods include smFISH, seqFISH, MERFISH/MERSCOPE, CosMx SMI, and Xenium; representative sequencing-based methods include Visium, Visium HD, Slide-seq, Stereo-seq, Seq-Scope, Curio Trekker, and GeoMx DSP. Shading distinguishes subcellular resolution (<10 µm) from resolution ≥10 µm.

ST platforms fall into two broad families that differ in their underlying chemistry (Table 1; Fig 1). Imaging-based (in situ hybridization) approaches detect individual transcripts in place using sequential rounds of fluorescent probe hybridization and imaging; representative methods include single-molecule FISH (smFISH), seqFISH, and MERFISH [8], with commercial implementations such as Vizgen MERSCOPE, NanoString CosMx SMI [9], and 10x Genomics Xenium [10]. Sequencing-based (spatial barcoding or capture) approaches capture transcripts on spatially barcoded arrays or beads and read them out by sequencing; representative methods include Visium and Visium HD [1,11], Slide-seq [12], Stereo-seq [13], Seq-Scope [14], the bead-array Curio Trekker, and the region-of-interest–based GeoMx Digital Spatial Profiler (DSP).

**Table 1:** Decision-support comparison of imaging-based (in situ hybridization) and sequencing-based (spatial barcoding/capture) spatial transcriptomics approaches. Resolution, coverage, tissue compatibility, availability, and cost are intended as orienting guidance; exact specifications vary by platform and version and should be verified against current vendor or method documentation before selection.

Feature/category	Imaging-based (in situ hybridization)	Sequencing-based (spatial barcoding/capture)
Representative methods	smFISH, seqFISH, MERFISH/MERSCOPE, CosMx SMI, Xenium	Visium, Visium HD, Slide-seq, Stereo-seq, Seq-Scope, Curio Trekker, GeoMx DSP
Principle	Transcripts detected in situ by sequential rounds of fluorescent probe hybridization and imaging	Transcripts captured on spatially barcoded arrays or beads and read out by sequencing
Spatial resolution	Subcellular (~100–500 nm)	Submicron to tens of µm depending on platform (e.g., Visium ~55/100 µm spots; Visium HD ~2 µm bins; Stereo-seq and Seq-Scope submicron)
Transcriptome coverage	Targeted panel (tens to a few thousand genes)	Genome-wide (whole transcriptome)
Quantification	High signal-to-noise; limited by probe hybridization efficiency and optical crowding	RNA-seq–like dynamic range; limited by capture efficiency and sequencing depth
Tissue compatibility	Platform-dependent; several support FFPE and/or fresh-frozen—confirm per platform [6]	Platform-dependent; Visium supports fresh-frozen and FFPE, several academic methods are fresh-frozen only—confirm per platform [6]
Commercial availability	Commercial (MERSCOPE, CosMx, Xenium) and academic (smFISH, seqFISH)	Commercial (Visium, Visium HD, Curio Trekker, GeoMx DSP) and academic (Slide-seq, Stereo-seq, Seq-Scope)
Indicative cost/accessibility	High instrument cost; cost scales with panel size and imaging time [6,16]	Cost scales with capture area and sequencing depth; academic methods can reduce reagent cost but require in-house expertise [6]
Typical use case	Hypothesis-testing on predefined gene sets at single-cell/subcellular resolution	Exploratory, genome-wide discovery of spatial programs
Key limitations	Panel must be predefined; custom probe design needed for new targets/organisms; optical crowding at high density	Spot/bead size may mix several cells; biases from capture efficiency and tissue permeability

The two families trade resolution against breadth in opposite directions. Imaging-based methods reach subcellular resolution and high sensitivity but typically profile a predefined panel of tens to a few thousand genes, suiting hypothesis-testing on targeted gene sets. Sequencing-based methods measure the transcriptome genome-wide, suiting exploratory discovery of uncharacterized spatial programs, but historically at coarser, multi-cell resolution. That gap is narrowing: Visium HD tiles ~2-µm capture areas toward near-single-cell resolution [11], Stereo-seq uses DNA-nanoball arrays at submicron spacing with adjustable bin size [13], and Seq-Scope exploits Illumina flow-cell clusters for submicron barcoding [14]. Resolution alone, however, does not determine the effective single-cell information recovered, which also depends on capture efficiency, transcript density, and segmentation quality (Tip 5).

Sample type often constrains platform choice as much as the biological question does, and these requirements are largely imposed by the platform rather than freely chosen. Academic platforms at high resolution (e.g., Stereo-seq, Seq-Scope) generally require fresh-frozen tissue, and thus prospective collection and snap-freezing. Probe- or capture-based targeted platforms (e.g., Visium FFPE, Xenium, CosMx) instead accept formalin-fixed paraffin-embedded (FFPE) material—making them applicable to archival and retrospective clinical cohorts—but, because FFPE RNA is fragmented and chemically modified, they depend on probe/capture chemistries rather than poly-A whole-transcriptome capture [6]. Identifying a platform’s required fixation and preservation conditions early, and aligning them with available samples, helps establish a feasible workflow and improves the reliability of the results [6]. Targeted platforms also differ in whether custom gene panels can be designed for a given tissue or organism, and some imaging-based assays have been explored for spatial detection of selected sequence variants; because this is platform- and assay-design-dependent, confirm that the specific variants, tissue, and probe design are supported before building variant detection into the design [15].

Finally, weigh commercial against noncommercial platforms. Commercial systems (e.g., Visium, Xenium, and CosMx) offer standardized reagents and support but carry substantial instrument and per-sample costs, whereas academic methods (e.g., Slide-seq, Stereo-seq, and Seq-Scope) can lower reagent costs or push resolution while demanding more in-house expertise [6]. The most expensive or highest-resolution platform is not automatically the best choice; recent independent benchmarks comparing commercial and academic ST platforms can inform this trade-off [6,16].

## Tip 3: Familiarize yourself with the structure of ST data before analysis

**Fig 2:**
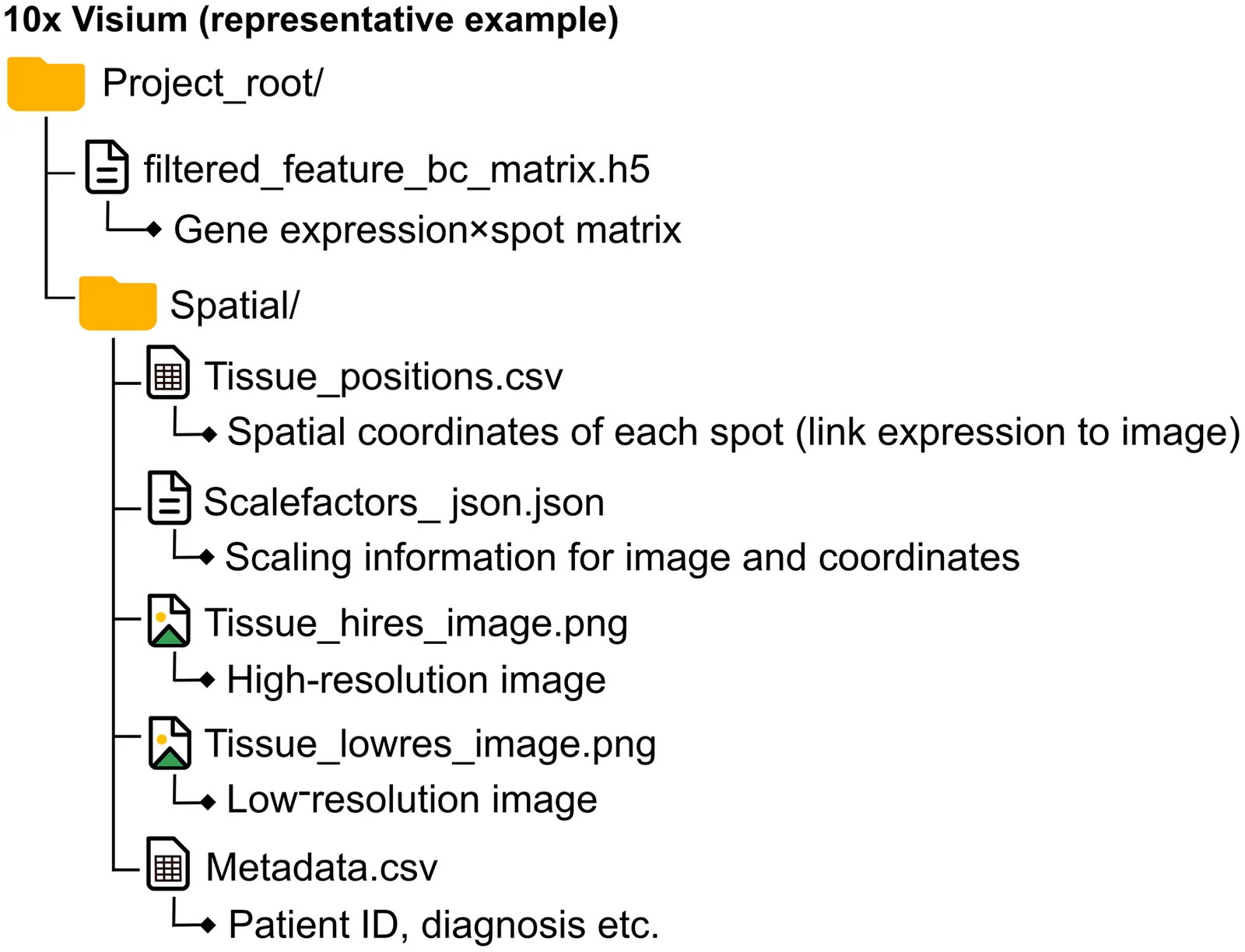
Directory structure of a 10x Visium spatial transcriptomics dataset, shown as a representative example. The project root contains the gene expression × spot matrix (filtered_feature_bc_matrix.h5) and a Spatial/subdirectory holding spatial coordinate files (Tissue_positions.csv), scaling parameters (Scalefactors_json.json), tissue images at multiple resolutions (Tissue_hires_image.png, Tissue_lowres_image.png), and sample metadata (Metadata.csv). Other platforms emit different files and conventions. Icons used in this figure were adapted from IconaMoon by Dariush Habibpour, obtained via Iconify, and are licensed under the Creative Commons Attribution 4.0 International License (CC BY 4.0). The icons were modified in color and appearance.

ST datasets comprise two distinct modalities: (i) tissue images (e.g., H&E or fluorescence) and (ii) spatially resolved gene-expression measurements linked to those images. Regardless of platform, most share a common set of components: an expression matrix of features (genes) by observations (spots, cells, or bins); spatial coordinates for each observation; feature/gene metadata; observation-level metadata; and one or more registered tissue images where available. The first analytical step is to generate the expression matrix of genes × spatial coordinates (spots or cells) from raw data (Fig 2): in sequencing-based methods by mapping reads that carry spatial barcodes, and in imaging-based methods by decoding multi-round fluorescence images.

The precise file organization is platform-specific. Taking 10x Visium as one common example, output is organized into a feature–barcode count matrix (e.g., filtered_feature_bc_matrix.h5) alongside a spatial directory holding tissue-position coordinates, scale factors, and tissue images at multiple resolutions (Fig 2). Other platforms—Slide-seq, Stereo-seq, Seq-Scope, or imaging-based assays—produce different files, so the Visium layout in Fig 2 is illustrative rather than a universal standard.

Where multiple cells occupy a single spot, image-based segmentation can estimate cell boundaries and reconstruct cell-level profiles [17]. When a reference scRNA-seq dataset is available (Tip 8), cell-type deconvolution can estimate the proportions of cell types within each spot [18].

For data management and reproducibility, standardized multi-layer formats—such as AnnData/H5AD (Python) [19] and Seurat objects (R) [20]—are recommended; these store the expression matrix, spatial coordinates, images, cell/gene metadata, and downstream results in a single file, facilitating integrated analysis and sharing.

## Tip 4: Follow a structured workflow to analyze ST data systematically

**Fig 3:**
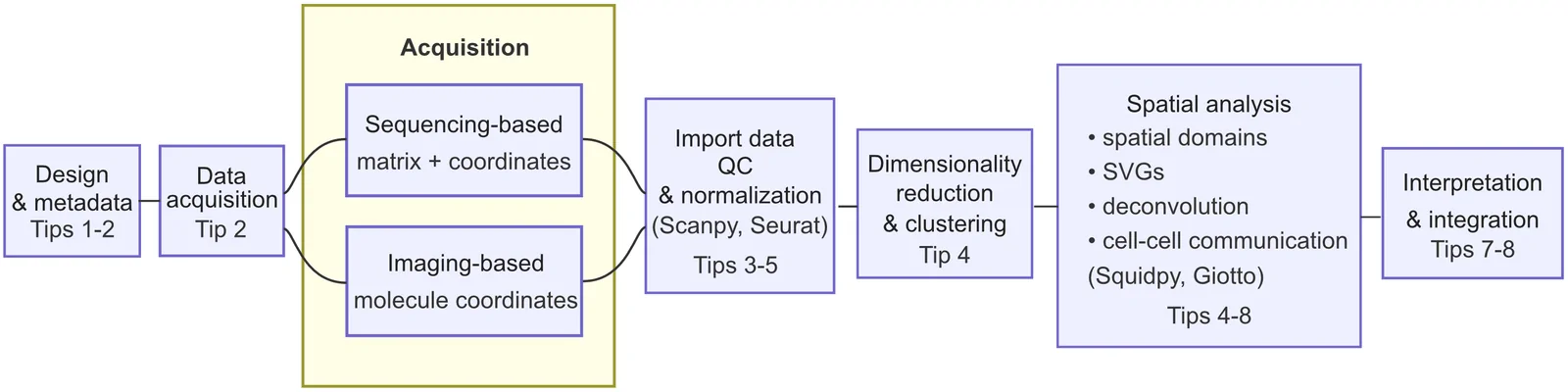
Spatial transcriptomics analysis workflow, drawn as a horizontal sequence of stages with representative software examples and the most relevant tips. Stages: (1) experimental design and metadata curation (Tips 1–2); (2) data acquisition by sequencing-based (capture) or imaging-based methods (Tip 2); (3) import, quality control, and normalization (Tips 3–5; e.g., Scanpy, Seurat, sctransform); (4) dimensionality reduction and clustering (Tip 4; e.g., Scanpy, Seurat); (5) spatial analysis—spatial-domain detection, spatially variable genes, deconvolution, and cell–cell communication inference (Tips 4–8; e.g., Squidpy, Giotto, SpatialDE, cell2location, NicheNet); and (6) biological interpretation and integration (Tips 7–8). Tool names are examples, not recommendations; see Table 2. Numbers in brackets indicate the most relevant tip(s).

Once the expression matrix is constructed, analysis proceeds through several stages that extend conventional scRNA-seq workflows with spatial information [20] (Fig 3). No single toolkit is required; those named below are examples, and Table 2 compares options so readers can choose by task rather than ecosystem.

**Table 2:** Representative software tools for spatial transcriptomics analysis, with supported analyses, a representative citation, and indicative strengths and limitations to aid selection. Tools are examples, not endorsements, and several alternatives exist for each task. Maintenance status, software versions, and current capabilities should be verified (e.g., on the project repository) at the time of use.

Tool (ecosystem)	Representative analyses	Citation (year)	Strengths	Limitations/notes
Seurat (R)	Single-cell/spatial integration, clustering, visualization	[20] (2021)	End-to-end workflow in R; broad documentation	General-purpose; spatial statistics less extensive than dedicated tools
Scanpy (Python)	Scalable single-cell/spatial preprocessing, clustering	[22] (2018)	Scales to large datasets; AnnData-based ecosystem	Spatial functions provided mainly via Squidpy
Squidpy (Python)	Spatial graphs, neighborhood enrichment, autocorrelation, ligand–receptor interaction	[25] (2022)	Purpose-built spatial statistics; integrates with Scanpy	Requires Scanpy/AnnData familiarity
Giotto (R)	Integrative spatial analysis and visualization	[30] (2021)	Wide range of spatial methods in one toolbox	Installation/dependency overhead
STUtility (R)	Visium-style processing, multi-section handling	[31] (2020)	Streamlines Visium workflows in Seurat	Oriented to capture-based data
BayesSpace (R)	Spatial Bayesian clustering, sub-spot resolution	[32] (2021)	Spatial priors enhance domain detection	Model assumptions; compute cost on large data
SpaGCN (Python)	Graph convolution over image, location, expression	[33] (2021)	Integrates histology with expression for domains	Parameter sensitivity
cell2location (Python)	Bayesian deconvolution of spot composition	[18] (2022)	Probabilistic per-spot cell-type estimates	Needs matched reference; GPU recommended
RCTD/spacexr (R)	Cell-type decomposition for spatial data	[34] (2022)	Handles multi-cell spots with reference profiles	Reference-dependent
Tangram (Python)	Mapping single cells to spatial coordinates	[35] (2021)	Flexible deep-learning alignment	Mapping quality depends on reference and tuning
DestVI (scvi-tools, Python)	Variational deconvolution/decomposition	[36] (2022)	Continuous within-type variation modeling	Training required
NicheNet (R)	Ligand→target gene communication inference	[24] (2020)	Links ligands to downstream target genes	Inference is hypothesis-generating, not causal (Tip 7)
LIANA (R/Python)	Unified ligand–receptor analysis	[37] (2022)	Consensus across multiple CCC methods	Inference, not proof of interaction
SpatialDE (Python)	Spatially variable gene detection	[23] (2018)	Statistical SVG identification	Compute cost; one of several SVG approaches
QuPath	Digital pathology image analysis	[28] (2017)	Whole-slide/digital pathology image analysis	Image-focused; not an expression-analysis suite
napari (Python)	Multi-dimensional image viewing/annotation	[29] (2019)	Extensible image viewer with plugins	Viewer/framework, not a full pipeline
Cellpose	Generalist deep-learning cell segmentation	[17] (2021)	Works across tissue/cell types	Segmentation errors propagate downstream (Tip 5)
PASTE/PASTE2	Slice alignment and 3D reconstruction	[38] (2022)	Optimal-transport alignment of adjacent sections	No method dominates across datasets (Tip 10)

### Preprocessing

Perform quality control (QC) to remove low-quality spots or cells (e.g., extremely low gene counts, abnormally high mitochondrial fractions), and normalize for differences in library size (total counts per spot). Because ST counts can be highly overdispersed, variance-stabilizing approaches such as sctransform (in Seurat) are often employed [21]. Because imaging-based and sequencing-based platforms generate counts differently, normalization and variance modeling should reflect platform chemistry, capture efficiency, panel design, and tissue type rather than be copied from scRNA-seq workflows.

### Core analysis

As in scRNA-seq, apply dimensionality reduction (e.g., principal component analysis) and clustering to partition the tissue into regions with similar expression profiles, characterizing tissue architecture and cellular distributions [20,22]. These steps ignore spatial coordinates and are therefore a starting point, not a spatial analysis.

### Spatially informed analysis

This is where ST’s strengths are fully leveraged. Representative analyses include identifying spatially variable genes (SVGs) with layered, gradient, or patch-like distributions [23]; inferring cell–cell communication from the spatial co-expression of ligands and receptors [24]; and detecting spatial domains (niches) via approaches such as neighborhood enrichment [25]. Others include spatial autocorrelation (e.g., Moran’s I), cell-type deconvolution of multi-cell spots against a reference (Tip 8), and segmentation-aware analysis tying expression to cell boundaries and histology (Tip 5). These reveal functional tissue units and aberrant intercellular signaling in disease. Because domain detection, SVG calling, and communication inference are each served by several methods with different assumptions (Table 2), results should be checked for robustness to method and parameter choice.

### Respect the statistical structure of the data

Spots or cells within a section are spatially autocorrelated and are not independent biological replicates; treating thousands of spots as the sample size inflates significance and constitutes pseudoreplication [26]. For group comparisons and differential expression, replication should be counted at the biological level (patient, animal, or sample), and spatial dependence modeled rather than ignored. Where conditions are confounded with slides or batches, observed differences can reflect technical rather than biological effects (Tip 5).

## Tip 5: Use dedicated tools for visualizing and segmenting spatial images

High-resolution tissue images in ST are invaluable, and inspecting histological context alongside gene-expression maps is indispensable. Platform vendors provide GUI applications, such as Loupe Browser (10x Genomics), that allow interactive visualization of gene expression overlaid on images without programming, enabling rapid assessment of data quality and gene localization.

Visual inspection is also the primary defense against tissue and image artifacts that distort downstream results. Watch for tissue folds, tears, detachment, and mounting or bubble artifacts. Watch also for regions of low RNA quality caused by degradation, suboptimal fixation, or—in fresh-frozen workflows—freezing and OCT-embedding damage. Sequencing-based assays additionally depend on permeabilization, which must be optimized for each tissue because over- or under-permeabilization biases capture [6,27]. Affected regions should be flagged or excluded during QC rather than interpreted as biology, and their distribution should be checked against slides and batches so that artifacts are not mistaken for condition effects (Tips 1 and 4).

For more advanced image analysis—such as machine-learning-based cell detection and the quantitative assessment of pathological features—open-source tools are powerful options. QuPath [28], a pathology image-analysis platform, and napari [29], a Python-based multidimensional image viewer, can support integration with ST data. Combining these environments with Cellpose, a general-purpose deep-learning-based segmentation tool, can help shift sequencing-based ST analysis from spot-level resolution toward single-cell-level spatial analysis by enabling the reconstruction of high-resolution cellular maps [17].

Segmentation quality depends on both the experiment and the analysis. Experimentally, nuclear and membrane staining determines how clearly cell boundaries appear in the image; computationally, deep-learning tools such as Cellpose [17] use those boundaries to assign transcripts to individual cells. When either step fails, cells are merged or split and transcripts are assigned to the wrong cell, and these errors carry through into per-cell expression estimates. Segmentation should therefore be inspected and validated rather than assumed to be correct, especially in dense or irregularly shaped tissue.

## Tip 6: Choose specialized software packages for ST gene-expression analysis

Effective analysis of ST gene-expression data typically relies on software packages designed for spatial information (Table 2). Such toolkits provide spatially aware data structures, specialized analytical algorithms, and visualization utilities, thereby substantially improving analytical efficiency and reproducibility. The right choice depends on the analysis at hand and the language a group already works in; Table 2 compares representative tools by supported analysis, strengths, and limitations.

In the R ecosystem, the scRNA-seq framework Seurat has integrated spatial analysis capabilities and is widely adopted [20], while Giotto [30] and STUtility [31] offer rich, ST-focused functionality. In Python, Scanpy [22] together with its extension Squidpy [25] is broadly used; Squidpy implements spatial statistics including construction of spatial graphs, neighborhood-enrichment analyses, and ligand–receptor interaction inference. Beyond these core packages, BayesSpace [32] incorporates spatial priors in a Bayesian framework to upsample spot-level data toward subspot resolution. As pipelines grow in complexity, encoding the analysis with workflow managers such as Nextflow or Snakemake is critical for ensuring reproducibility and facilitating collaboration.

## Tip 7: Ground your interpretation of spatial patterns in biological knowledge

The most important—and creative—stage of ST analysis is translating observed data patterns into coherent biological narratives. Heat maps and clustering gain scientific value only when anchored in biological meaning; interpreting the spatial localization of specific genes or the distribution of newly identified cell populations requires deep domain knowledge and systematic cross-referencing with the literature and relevant databases.

Several landmark studies exemplify this process. In Alzheimer’s disease, Chen *et al*. mapped gene expression around amyloid plaques and identified inflammatory programs in proximal glial cells [7]. In oncology, Ravi *et al*. integrated spatial transcriptomics and proteomics to reveal spatially segregated glioblastoma microenvironments with distinct metabolic and immune states [2]. In developmental biology, Asp *et al*. produced a spatiotemporal atlas of the human fetal heart, detailing cell-type distributions and gene-expression dynamics underlying structures such as the conduction system [4]. In each case, knowing where each measurement was taken—information that tissue dissociation discards—was essential to the insight. The shared lesson is to anchor interpretation in the tissue: align clusters and spatial domains to histology and anatomical landmarks and confirm domain identity with known marker genes before naming it.

Spatial proximity and inferred interaction also invite over-interpretation. Co-localization of two cell types, or co-expression of a ligand and its receptor, is consistent with interaction but does not by itself demonstrate that the cells communicate or that one state causes another; inferred cell–cell communication is best treated as hypothesis generation to be tested experimentally [24]. Therefore, hypotheses emerging from ST should, where possible, be validated against complementary data. Large language models (LLMs) have recently been explored for assisting cell-type annotation in single-cell omics [39]; while early results show moderate accuracy, such approaches require caution and expert oversight.

## Tip 8: Integrate ST with complementary omics data to deepen your findings

ST becomes more informative when integrated with complementary data. Integration with scRNA-seq is now routine: a reference atlas of cell types is mapped onto a section to estimate which cell types are present and in what proportions, giving cell-type-level interpretation of spot-based measurements rather than true single-cell resolution. Representative methods include cell2location [18], a Bayesian model of cell-type composition within each spot, and Tangram [35], which aligns single-cell profiles to spatial data with deep learning. Such integration is only as good as the reference: one that omits a cell type or derives from a different condition or technology can bias the mapping, so reference choice should match the tissue and question.

Beyond transcriptomes, ST can be combined with other spatial modalities for a multi-layered view of tissue. Spatial proteomics platforms such as CODEX or imaging mass cytometry (IMC) add protein-level readouts in the same frame, testing whether mRNA abundance tracks protein function [40,41]; DBiT-seq co-maps mRNA and protein from one section [42]. Emerging spatial epigenomic and metabolomic assays extend this to chromatin state and small-molecule distributions [43,44], complementary layers that transcriptomes alone cannot provide.

Integration is not automatically informative. Modalities differ in resolution, are often measured on different sections or platforms, and may require registration; resolution mismatch, batch effects, and alignment uncertainty can introduce artifacts that masquerade as biology. Before integrating, decide what each modality adds, verify registration, and confirm the combined analysis improves interpretation rather than merely adding complexity.

## Tip 9: Leverage public ST datasets and practice open science

ST is a rapidly evolving, interdisciplinary field in which cross-domain learning is highly beneficial. In neuroscience, the Allen Brain Map systematically relates brain structure to gene expression, providing gold-standard references and benchmarks for spatial analyses [45]; in developmental biology, a Stereo-seq-based atlas of *Drosophila* has produced three-dimensional spatiotemporal transcriptomic maps of embryonic and larval development, inspiring new analytical methods and concepts [46].

Many datasets are accessible through public repositories such as SpatialDB [47] and STOmicsDB [48] (Table 3), enabling preliminary hypothesis testing and method learning. Practicing open science—sharing code, metadata, and raw/processed data alongside manuscripts—enhances transparency and accelerates community-wide progress. For ST specifically, this means depositing raw and processed data (expression matrix, spatial coordinates, and tissue images) in standard, interoperable formats such as AnnData/H5AD; recording metadata on platform, tissue handling, and experimental design; sharing analysis code as versioned scripts or notebooks that state software versions and key parameters; and releasing data and code under permissive licenses that meet the target journal’s data-availability requirements so others can reanalyze and compare results.

**Table 3:** Public databases and resources for spatial transcriptomics data.

Name	Description	Official URL
SpatialDB	Database of spatial transcriptomics studies and datasets	https://www.spatialomics.org/
STOmicsDB	BGI/STOmics spatial data portal	https://db.cngb.org/stomics/
HuBMAP	Human BioMolecular Atlas Program (data & visualization)	https://hubmapconsortium.org/
Human Cell Atlas (HCA)	Human Cell Atlas data portal	https://www.humancellatlas.org/
BRAIN Initiative	US BRAIN Initiative portal	https://braininitiative.nih.gov/
Allen Brain Map	Allen Institute brain atlas and spatial data	https://portal.brain-map.org/
Vitessce	Web viewer for spatial & single-cell omics	https://vitessce.io/
10x Genomics Datasets	Official 10x public datasets collection	https://www.10xgenomics.com/resources/datasets

## Tip 10: Acknowledge the current technical limitations of ST

Like any technology, ST has current limitations that must be acknowledged to ensure rigorous interpretation.

Several limitations are technical and experimental. Sensitivity and detection efficiency are typically lower than bulk RNA-seq, so lowly expressed genes may be missed; spatial resolution varies by platform, and at multi-cell resolution each spot mixes signals from several cells, which deconvolution only partially resolves. Segmentation errors, tissue-processing artifacts (Tip 5), and batch effects between slides or runs further limit comparability, and cross-platform comparison remains difficult because each platform carries distinct biases [6]. Other limitations are statistical and computational: spatial autocorrelation and pseudoreplication complicate inference (Tip 4), and many spatial methods carry their own modeling assumptions and parameter sensitivity. Finally, ST is largely observational—co-localization and inferred interactions support hypotheses but rarely establish causation (Tip 7), so spatial associations should be framed as hypotheses unless supported by perturbation, orthogonal validation, or independent replication.

A further, much-discussed limitation is dimensionality: most ST methods rely on thin tissue sections and thus yield inherently two-dimensional measurements. Reconstructing true three-dimensional tissue architecture from serial sections introduces substantial technical challenges, and for experiments spanning many sections no universally accepted, high-precision method exists for aligning independently measured sections. In practice, researchers often combine multiple image-registration tools (e.g., Fiji/ImageJ, ANTs, and Elastix) and iteratively tune parameters by hand. Although nonrigid registration, optimal transport, and deep learning approaches have been proposed, heterogeneity in I/O formats, parameterization, and workflow integration has impeded robust, automated standardization, so many studies still analyze sections individually and qualitatively compare expression maps across sections.

Current algorithms such as PASTE [38] formulate alignment as an optimal-transport problem that balances transcriptional similarity with physical distance between adjacent sections. A recent benchmark of 12 multi-slice integration methods across 19 datasets found that no single method dominates universally; performance depends strongly on dataset characteristics, technology, and downstream goals [49]. These findings reinforce the need for context-aware method choice, transparent reporting of integration settings, and sensitivity analyses when reconstructing 3D tissue architecture.

## Conclusions

Spatial transcriptomics now provides a rapidly advancing framework for mapping gene expression in its native tissue context. This article consolidates practical guidance to help researchers formulate spatially explicit hypotheses, align platform selection and experimental design with those hypotheses, implement reproducible, spatially aware analytical workflows, and integrate orthogonal modalities to validate and extend findings. Because ST is observational, the associations it reveals should be treated as hypotheses. Adherence to these principles can help turn spatial maps into testable models of tissue organization and its perturbation in disease, validated with orthogonal and experimental data.
